# Characterization of Tear Immunoglobulins in a Small-Cohort of Keratoconus Patients

**DOI:** 10.1038/s41598-020-66442-7

**Published:** 2020-06-10

**Authors:** Tina B. McKay, Henrik Serjersen, Jesper Hjortdal, James D. Zieske, Dimitrios Karamichos

**Affiliations:** 1 Schepens Eye Research Institute/Massachusetts Eye and Ear, Department of Ophthalmology, Harvard Medical School, Boston, MA 02114 USA; 2 Department of Ophthalmology, Aarhus University Hospital, Aarhus, N DK-8200 Denmark; 3 North Texas Eye Research Institute, University of North Texas Health Science Center, Fort Worth, TX 76107 USA; 4 Department of Pharmaceutical Sciences, University of North Texas Health Science Center, Fort Worth, TX 76107 USA; 5 Department of Pharmacology and Neuroscience, University of North Texas Health Science Center, Fort Worth, TX 76107 USA

**Keywords:** Corneal diseases, Biomarkers

## Abstract

Keratoconus (KC) is classically considered a non-inflammatory condition caused by central corneal thinning that leads to astigmatism and reduced visual acuity. Previous studies have identified increased systemic levels of pro-inflammatory factors, including interleukin-6, tumor necrosis factor-α, and matrix metalloproteinase-9, suggesting that KC may have an inflammatory component in at least a subset of patients. In this study, we evaluated the levels of different immunoglobulins (light and heavy chains) based on Ig α, Ig λ, Ig κ, Ig µ, and Ig heavy chain subunits in non-KC tears (n = 7 control individuals) and KC tears (n = 7 KC patients) using tandem-liquid chromatography mass spectrometry. The most abundant Ig heavy chains detected in both control individuals and KC patients were Ig α-1 and Ig α-2 likely correlating to the higher IgA levels reported in human tears. We identified significant differences in immunoglobulin κ-chain V-II levels in KC patients compared to control individuals with no significant difference in Ig κ/Ig λ ratios or heavy chain levels. Our study supports previous findings suggesting that KC possesses a systemic component that may contribute to the KC pathology. Further studies are required to define causality and establish a role for systemic immune system-dependent factors and pro-inflammatory processes in KC development or progression.

## Introduction

Keratoconus (KC) is a corneal ectasia resulting from thinning of the corneal stroma that affects 1:375–1:2200 people worldwide^1–3^. Epidemiological studies have shown associations of KC with heightened immune response, such as atopic disease^4^, allergies^5^, and allergic rhinitis^2^ depending on the patient population. Furthermore, frequent eye rubbing remains a common behavior associated with KC likely contributing to poorer prognosis^6,7^. While previous studies have reported increased matrix metalloproteinase expression in KC tears^8,9^ and increased apoptosis of stromal keratocytes in KC corneas^10^, the underlying cause for stromal thinning remains relatively unknown. In terms of pathobiology, recent reports have suggested that KC is linked to elevated oxidative stress present in corneal fibroblasts^11^ and hypersensitivity to reactive oxygen species (ROS)^12,13^, which are both likely attributed to altered expression and activity of ROS-scavenging enzymes^14^. This increased susceptibility to ROS-induced cell death may contribute to loss of stromal cells within the cornea due to an inability to respond to environmental and internal cell stress.

Though KC is classically considered a non-inflammatory disease, a number of studies have identified increased expression of pro-inflammatory factors, such as interleukin (IL)-6 and tumor necrosis factor-α in KC tears^8^ and IL-16 and stem cell factor in saliva^15^. Increased Ig κ chain C and Ig J levels in KC tears have also been reported suggesting that B cell function may be altered in KC^16^. Early studies of serum samples from KC patients identified higher IgE^17^, IgG, and IgM levels in KC serum^4^ compared to controls likely consistent with a higher propensity for atopic disease in these patients. A more recent study showed downregulation of IgA tear levels that correlated to an overall reduction in total tear protein production and lactoferrin levels in KC patients^18^. However, a role of specific Ig classes in KC development or progression has not been clearly defined.

We have previously applied a quantitative proteomics approach to identify the presence of novel biomarkers present in KC tears, such as prolactin-inducible protein, lipophilin-A, immunoglobulin J chain, and cystatin-S^19^. The tear film is a useful biological fluid for characterizing systemic changes in secretions that may directly affect the ocular surface. Moreover, analysis of the tear film provides a unique opportunity to study early-mid stage KC patients to determine biochemical changes associated with disease progression. The tear film is generated by the combined efforts of the lacrimal gland, which provides most of the aqueous fluid, the meibomian gland, which is responsible for generating the lipid layer, and goblet cells within the conjunctiva that secrete the majority of the tear mucins^20^. The tear film is reported to be 3–40 μm thick in humans depending on the method of measurement ranging from filter paper collection to interferometry and confocal microscopy^21^. It is composed of salts and lipids, micronutrients, proteins, and water, all of which provide the required lubrication and nutritional support for the epithelial and conjunctival surfaces of the anterior segment. In terms of the functional effects of tears on the cornea, the interchange of certain growth factors present in the tear film, such as hepatocyte growth factor, transforming growth factor-β1, and platelet-derived growth factor, and the corneal epithelium and stroma may occur^22–24^. Thus, the components found in the tears may influence the cell phenotype of corneal epithelial cells, sensory neurons, and likely stromal keratocytes during corneal homeostasis and following wounding.

While B cells are commonly not found in the uninjured human cornea, immunoglobulins (Igs) are present in the tear film, which provides continual lubrication to the corneal surface, thus serving as the humoral immune response to inhibit microbial attachment or growth. The major heavy chains present in an antibody are defined by the Ig isotype. For example, Ig α compose the heavy chain of IgA antibodies, while Ig μ heavy chains are found in IgM antibodies. In contrast, the light chains, Ig λ and Ig κ are found in all of the major isotypes, IgG, IgA, IgM, IgD, and IgE. The dominant Ig isotype present in human tears is IgA, which is secreted from the lacrimal gland by acinar cells into the aqueous tear layer^25^. Igs may be isolated in human serum or tears with varying distributions depending on the method of analysis, e.g., free versus bound chains^26,27^. During B cell differentiation, rearrangement of the Ig genes is determined by a highly-regulated process known as V(D)J recombination^28^. The Ig κ/Ig λ ratio has been reported to be an important measure of B cell dysfunction, such as Ig light-chain amyloidosis and myeloma^29,30^. The normal ratio of Ig κ and Ig λ chains in serum has been reported to be 0.26–1.65^29,31^ with significantly lower Ig κ/Ig λ levels detected in tears (1.29 + 0.13) compared to serum (1.71 + 0.13)^32^. Significant changes in the relative ratios of Ig κ and Ig λ are associated with Ig light-chain amyloidosis and myeloma caused by abnormal antibodies produced by dysfunctional plasma cells^30^.

In this study, we evaluated the Ig distribution in KC tears compared to healthy controls using quantitative proteomics in order to determine if the relative ratio of heavy and light chains were altered with KC. To the authors’ knowledge, this study is the first to describe the Ig profile of heavy and light chain distributions and Ig κ/ Ig λ ratios in tears isolated from controls and KC patients. We identified significantly lower expression of select Ig κ light chains in KC patients compared to controls with no significant differential regulation of Ig heavy chains. Our novel findings support the growing evidence that KC possesses a systemic component that may contribute to the underlying pathobiology involved in corneal thinning.

## Materials and Methods

### Ethics statement

All experiments adhered to the Declaration of Helsinki. Patient consent was obtained from each patient prior to inclusion in the study. Institutional Review Board (IRB) approval (IRB Protocol #: 3450) from Dean McGee Eye Institute and Aarhus University Hospital (Approval # 1-10-72-77-14) was received according to institutional and federal guidelines. All tear samples were de-identified prior to analysis.

### Study design

**Figure 1:**
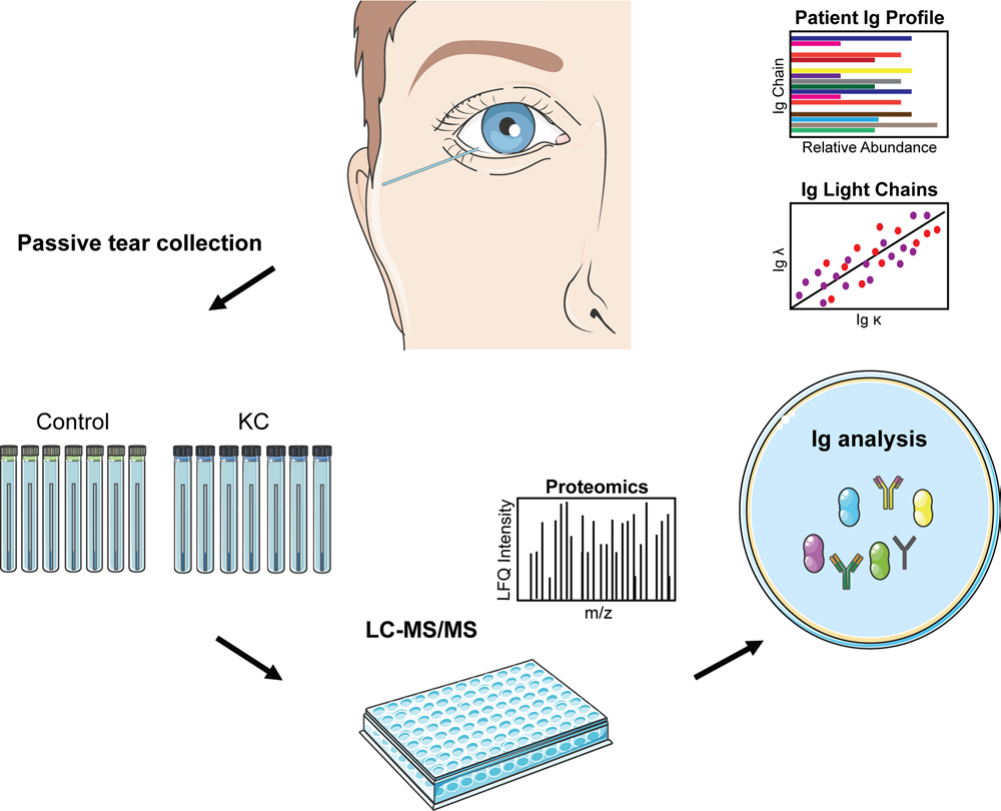
Experimental methodology for the characterization of Ig heavy and light chains detected in control and KC tears. Tears were collected from non-KC control individuals (n = 7) and KC patients (n = 7) using passive tear collection from the lateral tear meniscus into a glass capillary tube followed by proteomic analysis and immunoglobulin (Ig) characterization. The relative distributions of Ig α, Ig λ, Ig κ, and Ig µ were plotted for each individual. Pictorials modified from Servier Medical Art under a Creative Commons Attribution 3.0 Unported License available at smart.servier.com.

Given the close proximity of the tear film to the cornea, we evaluated the Ig protein contents contained within basal tears from non-KC control individuals and KC patients to determine if alterations in Ig heavy and light chains were associated with KC. We collected tear samples using a glass capillary oriented at the lateral tear meniscus to passively collect the tear film without promoting tear flux (Fig. 1). These tear samples were stored at −80 °C followed by proteomic analysis using unlabeled, quantitative liquid-tandem mass spectrometry. For this study, we analyzed the Ig chain distributions based on a population of non-KC control individuals and KC patients to determine the patient Ig profile and the relative differences in specific Ig subclass distributions.

### Inclusion and exclusion criteria

Healthy control individuals referred for refractive surgery with no clinical diagnosis of KC or ocular disease were included in the study (n = 7). The inclusion criteria for KC patients included a clinical diagnosis of KC by a certified ophthalmologist based on physical examination by slit lamp, refraction, best corrected visual acuity, and Pentacam HR Scheimpflug (Oculus, Wetzlar, Germany) tomography (n = 7). Any patients with evidence of other ocular diseases or infection were excluded from this study. We did not exclude control individuals or KC patients with reported or unreported allergies or atopic diseases.

### Tear collection

Passive tear collection was performed using a capillary tube (Hirchmann Laborgeräte, Germany) oriented at the lateral tear meniscus with the isolation of ~5 µL of tears per patient. The capillary tubes were immediately transferred to a sealed test tube and stored at −80 °C until further analysis. Transfer of the sample from the capillary tube was performed using a micropipette.

### Proteomics analysis

Based on previously established protocols^19,33^, tear samples were analyzed using an EASY-nLC nanoflow HPLC with a 75 µm inner diameter × 15 cm length C18 capillary column coupled to a hybrid LTQ Orbitrap XL-ETD mass spectrometer (Thermo Fisher Scientific, Waltham, MA) using label-free quantification and analysis. Further analysis was performed on immunoglobulin chains detected in at least 3 patients. All values detected in at least 3 patients was evaluated with no omission of samples with (0) or missing values.

### Statistical analysis

GraphPad Prism 7 for Windows (GraphPad Software, Version 7.03, La Jolla, CA, USA, www.graphpad.com) was used for statistical analysis. Statistical significance for comparing multiple groups was determined based on a two-way ANOVA with Sidak’s multiple comparisons test and α set to 0.05. A p < 0.05 was considered significant.

## Results

For our study, we evaluated the distribution of heavy and light chains detected in human tear samples from control individuals and KC patients (Table 1). For each subclass (e.g., Ig α, Ig λ, Ig κ, Ig μ, and Ig heavy chain), select fragments were detected and identified based on certain regions of the protein (for example – chain C, chain V-IV, chain V-I, among others). The most abundant subclass detected in control individuals was Ig κ chain V-II with an average expression of 1.23E + 09 (±1.60E + 09) LFQ (label-free quantification) intensity detected in 43% of patients (Table 1). In contrast, Ig α-1 was the most abundant subclass detected in KC patients (6.26E + 08 + 6.78E + 08 LFQ intensity) with a similar expression pattern as that found in control individuals (6.76E + 08 + 6.90E + 08 LFQ intensity) (Table 1).

**Table 1:** The distribution of immunoglobulin (Ig) heavy and light chains detected in tears isolated from control individuals and KC patients.

Ig Factor	Chain	Ig Isotype	Control Individuals		KC Patients	
			Relative abundance + Stdev (LFQ Intensity)	% of patients with detectable expression	Relative abundance + Stdev (LFQ Intensity)	% of patients with detectable expression
Ig α-1 chain C	heavy	IgA	6.76E + 08± 6.90E + 08	57%	6.26E + 08± 6.78E + 08	57%
Ig α-2 chain C	heavy	IgA	4.02E + 08± 5.41E + 08	100%	2.32E + 08± 4.21E + 08	71%
Ig λ-7 chain C	light	IgG, IgA, IgM, IgD, IgE	7.00E + 04± 1.85E + 05	14%	6.51E + 05± 1.48E + 06	43%
Ig λ chain V-IV	light	IgG, IgA, IgM, IgD, IgE	1.89E + 06± 2.37E + 06	43%	1.03E + 06± 1.77E + 06	29%
Ig κ chain C	light	IgG, IgA, IgM, IgD, IgE	1.21E + 08± 1.21E + 08	71%	3.62E + 07± 5.29E + 07	43%
Ig κ chain V-I	light	IgG, IgA, IgM, IgD, IgE	2.37E + 07± 6.26E + 07	14%	5.00E + 06 + 6.88E + 06	43%
Ig κ chain V-II	light	IgG, IgA, IgM, IgD, IgE	1.23E + 09± 1.60E + 09	43%	2.35E + 08 + 3.22E + 08	71%
Ig κ chain V-III (a)	light	IgG, IgA, IgM, IgD, IgE	1.74E + 06± 1.71E + 06	57%	0.00E + 00± 0.00E + 00	0%
Ig κ chain V-III (b)	light	IgG, IgA, IgM, IgD, IgE	2.95E + 07± 7.31E + 07	29%	5.15E + 06± 5.99E + 06	57%
Ig κ chain V-III (c)	light	IgG, IgA, IgM, IgD, IgE	1.64E + 07± 3.89E + 07	71%	0.00E + 00± 0.00E + 00	0%
Ig κ chain V-IV	light	IgG, IgA, IgM, IgD, IgE	6.07E + 05± 1.61E + 06	14%	1.97E + 06± 2.51E + 06	57%
Ig μ chain C	heavy	IgM	3.19E + 07± 3.05E + 07	100%	4.66E + 07± 5.97E + 07	100%
Ig heavy chain V-I	heavy	IgG, IgA, IgM, IgD, IgE	1.06E + 06 + 1.37E + 06	43%	1.38E + 05± 3.65E + 05	14%
Ig heavy chain V-II	heavy	IgG, IgA, IgM, IgD, IgE	0.00E + 00± 0.00E + 00	0%	4.64E + 05± 6.01E + 05	43%
Ig heavy chain V-III (d)	heavy	IgG, IgA, IgM, IgD, IgE	0.00E + 00± 0.00E + 00	0%	1.05E + 06± 1.49E + 06	43%
Ig heavy chain V-III (e)	heavy	IgG, IgA, IgM, IgD, IgE	4.58E + 06± 6.31E + 06	43%	5.00E + 06± 6.26E + 06	57%

Specific regions identified separately within the same chain are reported here: (a) Ig κ chain V-III region VG; (b) Ig κ chain V-III region HAH; Ig κ chain V-III region HIC; Ig κ chain V-III region SIE; Ig κ chain V-III region WOL; Ig κ chain V-III region Ti; Ig κ chain V-III region GOL; (c) Ig κ chain V-III region CLL; Ig κ chain V-III region VH; Ig κ chain V-III region POM;Ig κ chain V-I region Lay; (d) Ig heavy chain V-III region VH26; (e) Ig heavy chain V-III region BRO;Ig heavy chain V-III region TEI;Ig heavy chain V-III region BUT;Ig heavy chain V-III region WEA.

**Figure 2:**
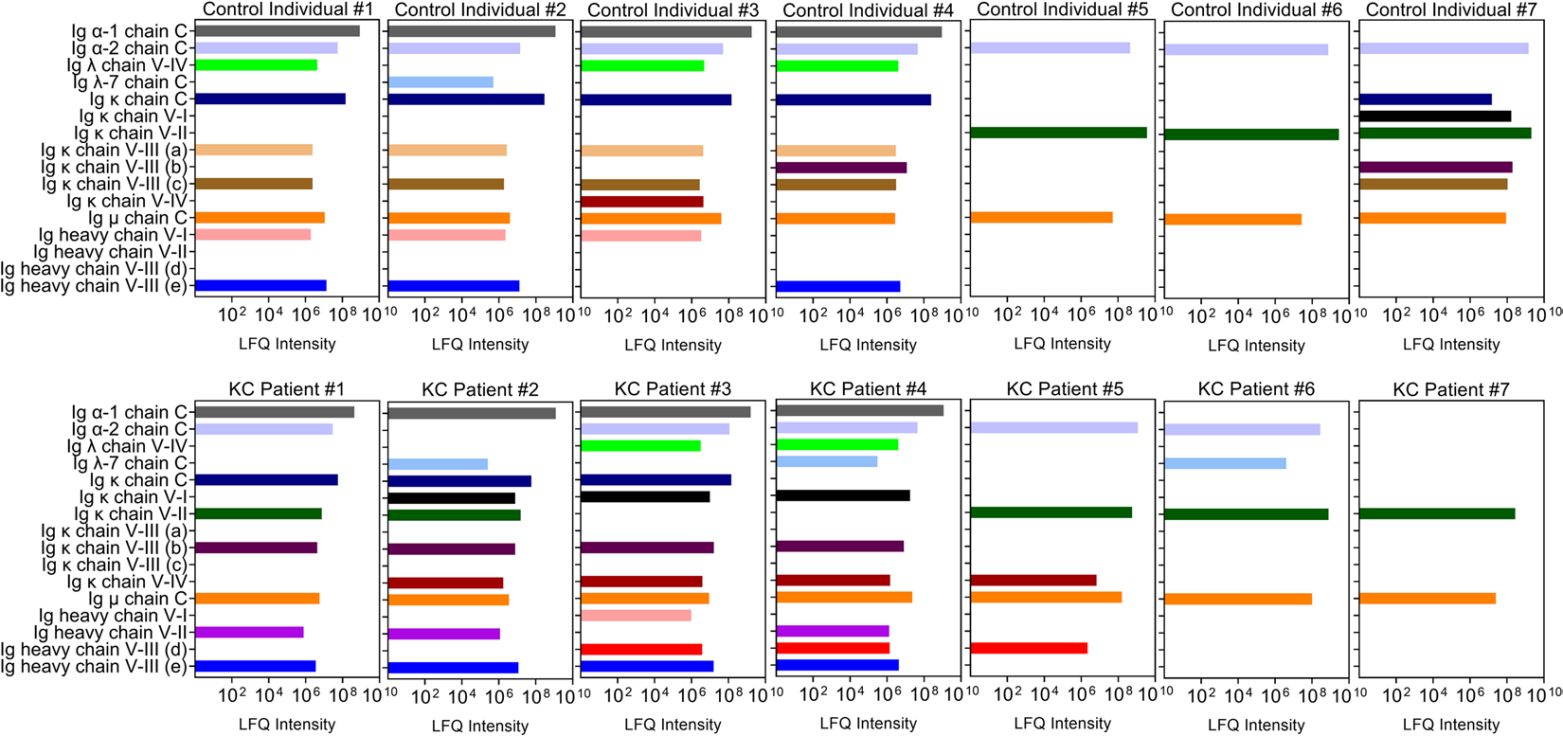
Patient immunoglobulin (Ig) profile identified in tears isolated from non-KC control individuals and KC patients determined by quantitative proteomic analysis. The relative distributions of Ig α, Ig λ, Ig κ, Ig µ, and heavy chain were plotted for each individual. (a–e) Refer to Table 1 for the description of the regions included in the different species.

We evaluated the Ig profile for each patient to determine if trends existed within the control and KC populations. The major heavy chain subclasses, Ig α-2 and Ig μ, showed little variability between patients with consistent expression in most samples (Fig. 2). Interestingly, the heavy chain Ig μ was detected in every patient sample with similar expression levels from patient to patient and between groups (control versus KC) (Fig. 2). Moreover, certain Ig chains were detected only in KC samples, including Ig heavy chain V-II and Ig heavy chain V-III (d), which were both absent in all control samples (Fig. 2). In contrast, Ig κ chain V-III (a) was only detected in four of seven control individuals with no expression found in KC patients. While Ig κ chain V-II was identified in most KC patients (71%), the relative expression level appeared lower than that found in certain control individuals (controls #5, #6, #7) (Fig. 2).

**Figure 3:**
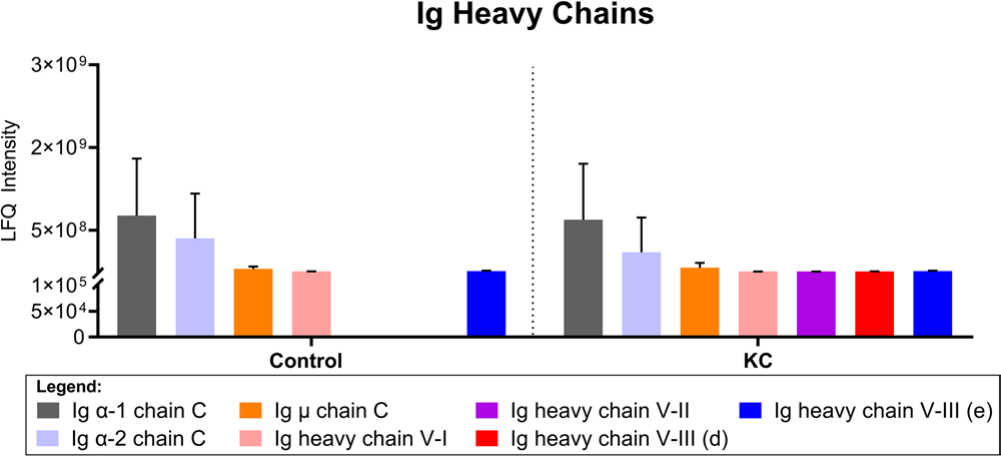
Comparative analysis of immunoglobulin (Ig) heavy chains (Ig α, Ig μ, and Ig heavy chain V-1, V-II, and V-III) in tears isolated from non-KC control individuals and KC patients. The error bars depict the standard deviation. All patient samples were included in the analysis. Statistical analysis determined based on a two-way ANOVA with Sidak’s multiple comparisons test and α set to 0.05. (d and e) Refer to Table 1 for the description of the regions included in the different species.

In order to determine if collective differences in Ig chains existed between control individuals and KC patients, we evaluated the relative distribution of heavy chain subclasses (Ig α, Ig μ, and Ig heavy chain V-I, V-III) detected in tear samples from each group. We identified no significant difference in Ig heavy chain distribution between control individuals and KC patients with certain chains showing no expression in controls (Ig heavy chain V-II and Ig heavy chain V-III (a)), though with similar variability in KC patients (Fig. 3). Likewise, the most abundant heavy chain detected in the tears (Ig α-1 and Ig α-2) showed similar expression levels in control individuals and KC patients (Fig. 3).

**Figure 4:**
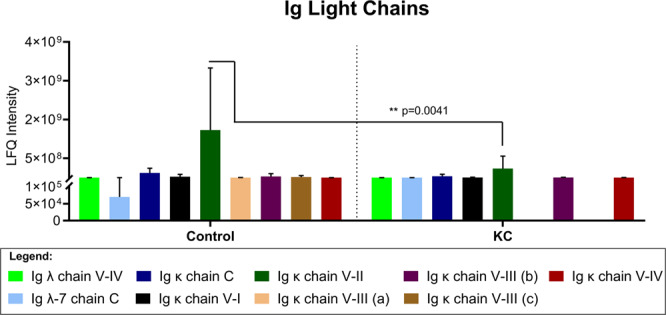
Comparative analysis of immunoglobulin (Ig) light chains (Ig λ and Ig κ) detected in tears from non-KC control individuals and KC patients. The error bars depict the standard deviation. All patient samples were included in the analysis. (**a–c**) Refer to Table 1 for the description of the regions included in the different species. Statistical analysis determined based on a two-way ANOVA with Sidak’s multiple comparisons test and α set to 0.05.

To determine if Ig light chains showed a similar pattern in controls and KC patients, we plotted the relative distribution of each Ig chain identified in the tear samples. A number of Ig light chains showed relatively uniform expression between groups, including Ig λ chain V-IV, Ig λ-7 chain C, Ig κ chain C, Ig κ chain V-I, Ig κ chain V-III (b), and Ig κ chain V-IV (Fig. 4). However, three Ig κ light chains, e.g., Ig κ chain V-II, Ig κ chain V-III (a), and Ig κ chain V-III (c), were consistently lower in KC samples compared to controls (Fig. 4). Ig κ chain V-II was found to be significantly higher on average (521%, p = 0.0041) in control individuals compared to the KC group (Fig. 4).

**Figure 5:**
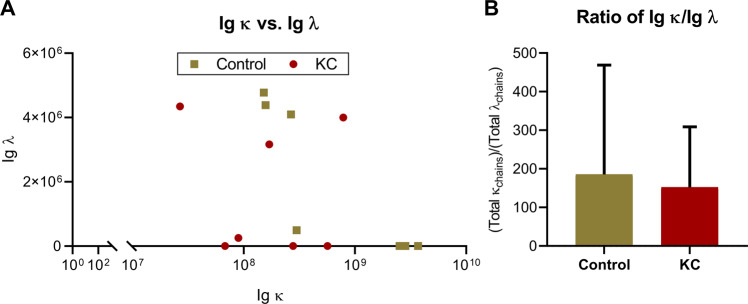
The relative distribution of total immunoglobulin (Ig) κ and Ig λ light chain ratios detected in non-KC control individuals and KC patients. (**A**) The ratios of Ig κ versus Ig λ are shown. Units for each light chain correspond to the LFQ intensity detected. (**B**) The relative ratio of of Ig κ and Ig λ were determined based on the total subtypes detected by proteomic analysis. No Ig λ was detected in (3) control and (3) KC tear samples and thus were excluded from this calculation. The error bars represent standard deviation.

Given a potential role for changes in light chain levels and specific diseases associated with B cell function, we assessed the relative ratio of Ig κ and Ig λ to determine if the lower Ig κ levels found in certain KC patients gave rise to differential light chain distributions (Fig. 5). We found an apparent inverse relationship between Ig κ and Ig λ levels in certain control samples with high Ig κ levels corresponding to no detectable Ig λ levels (Fig. 5A). In terms of Ig κ/Ig λ ratios, both controls and KC samples showed a similar distribution with no signficant difference between groups (Fig. 5B).

## Discussion

In this study, the most abundant Ig heavy chains detected in both control and KC tears was Ig α-1 and Ig α-2 consistent with previous studies showing higher IgA compared to IgG or IgM in tears^34,35^. In contrast to a previous study showing elevated Ig κ chain C^16^, we identified no significant difference in this subclass in our study but identified significantly decreased Ig κ chain V-II levels. Moreover, the downregulation in Ig κ levels in KC tears appeared to be consistent for other detected Ig κ chains (e.g., Ig κ chain V-III) within the small KC population analyzed in this paper. Heavy chain distributions (Ig α and Ig μ chains) were unaltered between control individuals and KC patients suggesting that the variations in light chains may be related to altered light chain distributions during B cell development, rather than differences in isotype concentrations between groups.

Recent reports have suggested that systemic factors may play a contributory role in KC development and thus may serve as potential biomarkers for diagnosis or possible modes for therapeutic intervention^36^. Altered levels of different hormones (e.g., dehydroepiandrosterone-sulfate, estrone, cortisol, prolactin, and thyroxine) have been detected in KC saliva^15,37^, plasma^37^, hair follicles^38^, aqueous humor^39^, and tears^40^, respectively. Given the known interplay between hormones and immune cell function^41,42^, the influence of hormones on corneal thickness in the context of KC development and progression remains a growing area of interest.

While this study identified significant variations in Ig κ levels in KC tears, limitations in the relative small sample size in both the controls and KC populations may contribute to lower confidence in the applicability of the findings to the larger patient population. Moreover, parallel evaluation of Ig levels in tears and serum, as well as assessment of intact antibody levels (IgG versus IgA) are warranted to determine if a potential role for altered B cell regulation is present in KC. Further studies analyzing blood or saliva levels are warranted to determine whether systemic changes in Ig levels occur in KC. While utilization of rigorous, high-throughput approaches, such as proteomics, provides significant amounts of information regarding a sample (protein identity and relative abundance), the lack of detection of certain proteins likewise occurs giving rise to missing values, e.g., data values of 0. However, the advantages of using mass spectrometry over traditional antibody-based approaches, such as an enzyme-linked immunosorbent assay, include no restrictions on detection that are dependent on antibody recognition and binding efficiency.

Using protein microarrays, we have previously characterized a panel of pro-inflammatory factors, including Ig light chains, in saliva isolated from healthy and KC patients^15^. Consistent with the current findings, we identified no significant difference in the total salivary Ig light chain abundance in healthy versus KC samples [0.053 ± 0.038 (n = 6) versus 0.086 ± 0.053 (n = 8), respectively, p = 0.2]^15^. The results from the work presented in this paper using tear samples and proteomics, which is able to distinguish between different subtypes of light and heavy chains, suggest that Ig κ may be selectively regulated in KC. In this paper, we reported proteomic analysis of 7 patient tear samples per group with the detected Ig heavy and light chains that were identified in at least 3 patient samples, thus allowing inclusion of Igs identified with high confidence in the detection of the assigned Ig chain. Our results suggest that Ig light chain distributions in tears may be altered in at least a subset of KC patients. Defining causation in terms of KC development over simple association of elevated Ig κ levels may be difficult due to the heterogeneity of the patient population, lack of an animal model, and limited mechanistic insight into the physiological role of these factors in the tear film. Further validation of our findings are required in a larger control and KC patient population to exclude any contributions from allergic conditions. Allergic and atopic diseases have been previously associated with KC^2^. In our study, we did not exclude the presence of these immune conditions from our analysis. However, the levels of IgA and IgM heavy chains were not significantly different between control individuals and KC patients suggesting that the differences in Ig κ-chain V-II levels may be independent of an overt allergic condition. Further studies are warranted to clarify the role of the immune system and other systemic factors in the context of KC development or progression.

## Supplementary information

Supplementary Information.

## Data Availability

The datasets analyzed in the current study are available from the corresponding author upon request.
