# Supplementary material for: Characterization of Tear Immunoglobulins in a Small-Cohort of Keratoconus Patients
Source: Sci Rep. 2020 Jun 10;10:9426. doi: 10.1038/s41598-020-66442-7 (PMC7287105; doi:10.1038/s41598-020-66442-7)
Supplement: Supplementary file 1 — Supplementary Information. [file 41598_2020_66442_MOESM1_ESM.pdf]

Characterization of Tear Immunoglobulins in a Small-Cohort of Keratoconus Patients

Tina B. McKay[1], Henrik Serjersen[2], Jesper Hjortdal[2], James D. Zieske[1], and Dimitrios Karamichos[3,4,*]

[1]Schepens Eye Research Institute/Massachusetts Eye and Ear, Department of Ophthalmology, Harvard Medical School, Boston, MA, 02114, USA

[2]Department of Ophthalmology, Aarhus University Hospital, Aarhus N DK-8200, Denmark

[3]Department of Ophthalmology/Dean McGee Eye Institute, University of Oklahoma Health Sciences Center, Oklahoma City, OK 73104, USA

[4]Department of Cell Biology, University of Oklahoma Health Sciences Center, Oklahoma City, OK 73104 USA

*Corresponding Author: Dimitrios Karamichos, Ph.D., Department of Ophthalmology/Dean McGee Eye Institute, University of Oklahoma Health Sciences Center, Oklahoma City, OK 73104; Phone: (405) 271 4019; FAX: (405) 271 8128; email: dimitrios-karamichos@ouhsc.edu

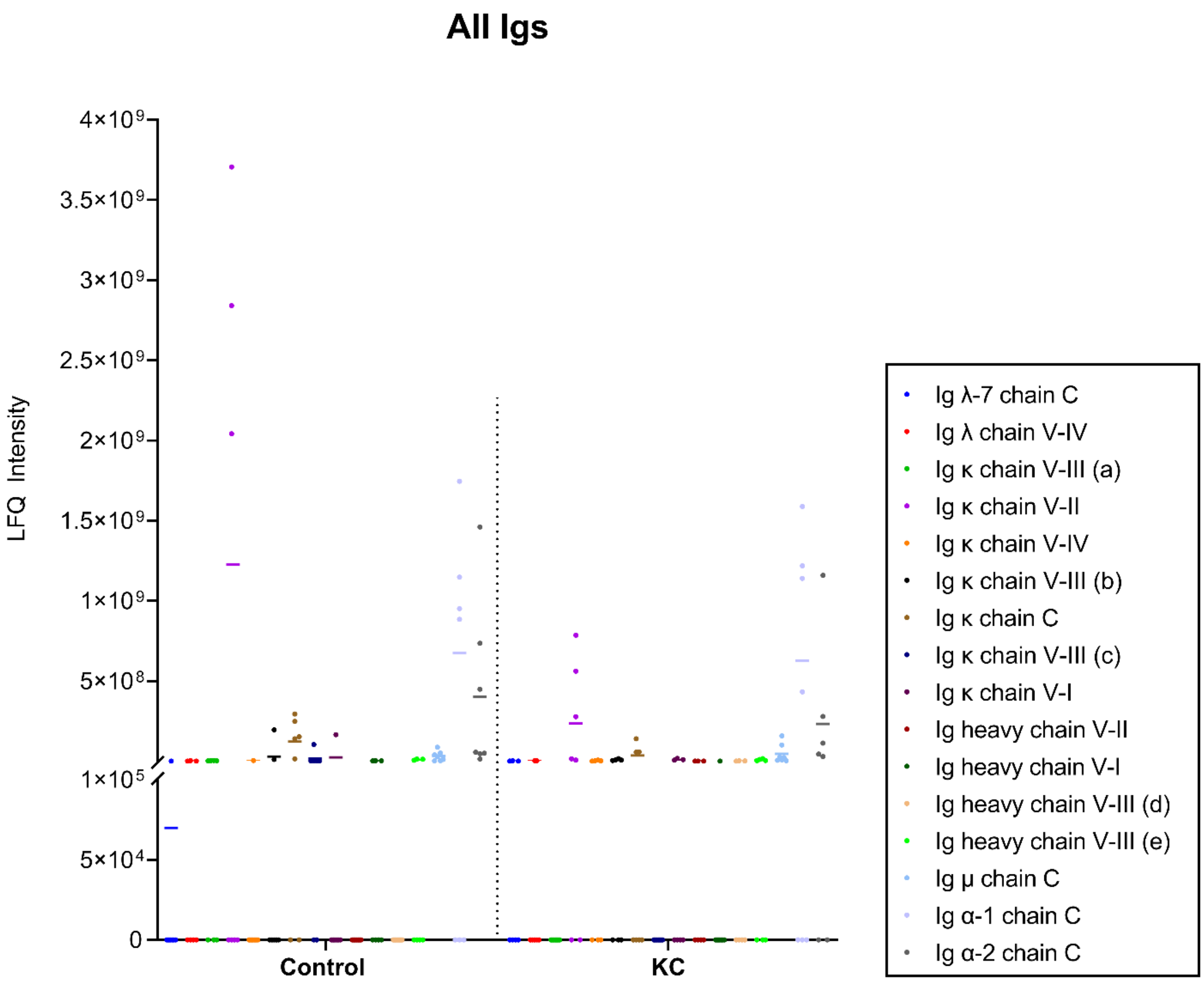

Supplemental Fig. 1. Comparative analysis of immunoglobulin (Ig) chains showing individual control and KC samples detected by proteomics.
